# Hypercoagulability State Combined with Post-Treatment Hypofibrinolysis in Invasive Breast Cancer: A Seven-Year Follow-Up Evaluating Disease-Free and Overall Survival

**DOI:** 10.3390/life13051106

**Published:** 2023-04-28

**Authors:** Katarzyna Wrzeszcz, Piotr Rhone, Katarzyna Kwiatkowska, Barbara Ruszkowska-Ciastek

**Affiliations:** 1 Department of Pathophysiology, Faculty of Pharmacy, Nicolaus Copernicus University, Collegium Medicum, 85-094 Bydgoszcz, Poland; katarzynakwiatkowska@abs.umk.pl; 2 Clinical Ward of Breast Cancer and Reconstructive Surgery, Oncology Centre Prof. F. Łukaszczyk Memorial Hospital, 85-796 Bydgoszcz, Poland; rhonep@co.bydgoszcz.pl

**Keywords:** breast cancer, adjuvant treatment, hemostasis, cancer therapy-associated thrombosis

## Abstract

(1) Background: Cancer treatment, including chemotherapy, endocrine therapy, targeted therapy and radiotherapy, has been identified as an important independent risk factor for venous thromboembolism in cancer patients. The aim of the study was to evaluate the effect of adjuvant therapy on the coagulation and fibrinolysis components in invasive breast cancer. (2) Methods: Tissue factor pathway inhibitor (TFPI), tissue factor (TF), tissue plasminogen activator (t-PA), plasminogen activator inhibitor-1 (PAI-1) antigen (concentration) and TFPI and TF activities were examined in the blood samples of 60 breast cancer patients treated by adjuvant chemotherapy, endocrine therapy, radiotherapy and immunotherapy. Blood samples were taken 24 h before primary surgery and 8 months after tumour removal surgery. (3) Results: Adjuvant therapy administrated to breast cancer patients significantly increased the concentration of plasma TF, the PAI-1 antigen and also the activity of TFPI and TF, but significantly decreased the level of the t-PA antigen. Combined chemotherapy and endocrine therapy, but not monotherapy, has an important effect on haemostatic biomarker levels. (4) Conclusions: Breast cancer patients receiving adjuvant therapy have an elevated risk of developing a hypercoagulability and hypofibrinolysis state leading to venous thromboembolism.

## 1. Introduction

Breast cancer treatment is multidisciplinary, and the main factors determining the therapy decision-making depend on the stage and biology of the tumour [1,2]. The individualised plan for the clinical management of breast cancer currently still relies on traditional prognostic and predictive factors, including clinical, histological and some well-defined biological factors such as hormone receptors: ER (oestrogen receptor), PR (progesterone receptor) and HER2 (human epidermal growth factor receptor 2) expression [3]. Adjuvant therapy, given in addition to primary surgery, plays a critical role in the treatment of early-stage breast cancer. It may consist of local treatment (radiotherapy) and systemic treatments (chemotherapy, endocrine and biological therapies). Recent advances in therapeutic patterns have developed more precise delivery of adjuvant treatment with an improvement in survival rates by reducing the risk of local and distant recurrence [4]. Despite the effectiveness of contemporary anticancer therapies, there are significant potential adverse effects. Of particular concern, multiple cancer therapies are associated with an increased risk of thrombosis [5]. Cancer treatments, including chemotherapy, endocrine therapy, targeted therapy and radiotherapy, have been identified as an important independent risk factor for VTE (venous thromboembolism) in cancer patients [6].

Haemostasis is a fundamental process in the maintenance of circulation, involving an intricate, highly balanced interaction between blood vessels, platelets, plasma coagulation factors and fibrinolytic proteins in the formation and dissolution of blood clots [7,8]. Imbalance between coagulation and the fibrinolysis process initiates and promotes thrombosis, which is one of the leading causes of death in cancer [9]. The pathomechanism of blood coagulation and fibrinolysis activation in cancer is complex and multifactorial and its exact pathophysiological pathways are still under elucidation [10]. There are multiple pathophysiological processes involved in the interplay between cancer and various components of the haemostatic system [11]. One of the key factors contributing to the development of haemostatic disorders in cancer is the tumour-cell-associated clot promotion consisting of the production of procoagulant factors by tumour cells [10]. Of these, tissue factor (TF) overexpressed on the malignant cell surface can play a crucial role in cancer-induced coagulation, resulting in clotting cascade activation. A high TF expression in the tumour tissue of cancer patients has been correlated with tumour progression, worse clinical outcomes and thrombosis [12]. Other principal pathways of cancer-associated thrombosis include the tumour-cell expression of fibrinolysis proteins—urokinase-type plasminogen activator (u-PA), tissue-type plasminogen activator (t-PA) and plasminogen activation inhibitor-1 (PAI-1)—and cytokines interleukin-1 (IL-1) and tumour necrosis factor alpha (TNF-α). As the fibrinolytic factors are primarily involved in the breakdown of fibrin, u-PA and t-PA, their proteolytic properties also enable the activation of metalloproteinases resulting in disintegration and degradation of the extracellular matrix, which in turn promotes neoplastic cell migration and tumour invasion. The potential interaction of tumour-overexpressed inflammatory cytokines with the haemostatic system involves thrombogenicity promotion and fibrinolysis inhibition through inducing TF and PAI-1 expression by endothelial cells [13,14].

Additional mechanisms of blood-clotting activation in the course of cancer are triggered by the antineoplastic medications. Anticancer drugs are capable of inducing thrombogenic effects through multiple different mechanisms: (1) release of procoagulants and proinflammatory cytokines from damaged neoplasm cells; (2) direct drug toxicity on the vascular endothelium; (3) direct induction of monocyte or tumour-cell expression of TF; (4) a decrease in physiological anticoagulants; and (5) platelet activation and aggregation. The direct injury of endothelial cells by anticancer agents leading to TF overexpression and a loss of antithrombotic properties appears to be the most important cause of cancer therapy-associated thrombosis. Though current anticancer treatment is an effective and reliable way to treat many types of cancer, patients receiving adjuvant therapy are at an elevated risk of thrombosis and recurrent events independent of their neoplasm. Several cytotoxic chemotherapeutic agents, as well as endocrine therapy, radiotherapy and targeted therapy, have demonstrated side-effects on the haemostasis process [13,15,16].

Even though there is a plethora of encouraging clinical evidence on the potential impact of anticancer therapy on haemostatic parameters studied in different kinds of cancers, surprisingly this interaction in the early stage of invasive breast cancer (IBrC) has been not fully evaluated. In this seven-year prospective, single-institution study, the components of procoagulant, anticoagulant and fibrinolytic system were investigated before and after the administration of adjuvant treatment to assess the effects of the potential impact of adjuvant therapy on future clinical outcomes with respect to disturbance of the haemostatic balance in women with breast cancer.

## 2. Materials and Methods

### 2.1. Study Participants and Design

**Figure 1:**
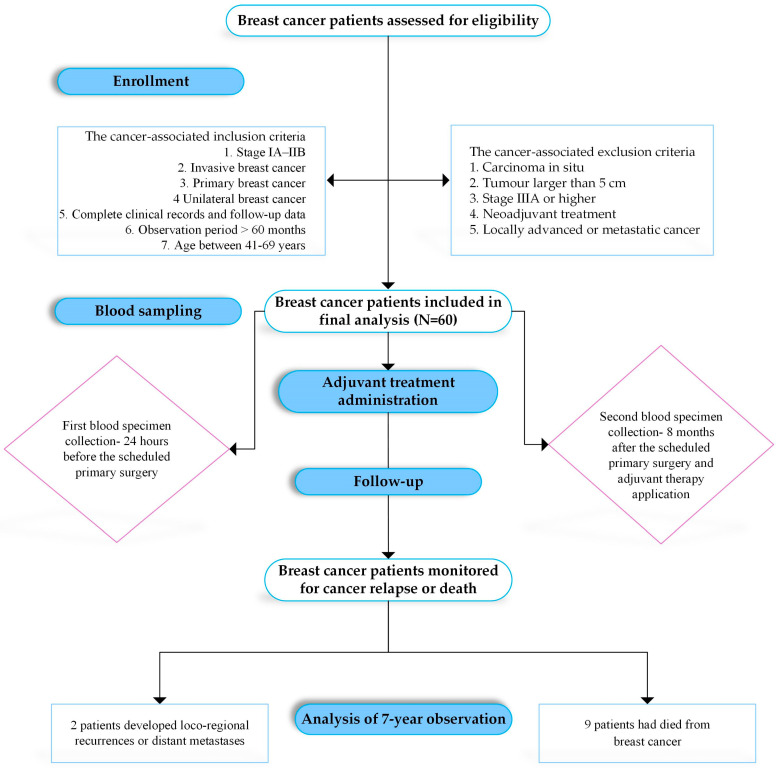
Flowchart of the study.

A total of 60 primary IBrC patients diagnosed between November 2015 and June 2017 at the Clinical Ward of Breast Cancer and Reconstructive Surgery, Oncology Centre in Bydgoszcz, Poland were eligible for this study. Clinical information was obtained from medical records based on interviews conducted by an oncologist with expertise in breast cancer. The study protocol was approved by the Bioethics Committee of the Nicolaus Copernicus University in Toruń at Collegium Medicum im. Ludwik Rydygier in Bydgoszcz (reference number: KB/547/2015). Clinical data were collected in accordance with the guidelines laid down in the Declaration of Helsinki of 1975, as revised in 2000. Patients’ written informed consent to the use of their data and biological material was sought prior to enrolling in the study. Traditional methods for breast cancer diagnosis, including histological and clinical factors and molecular marker analysis, were provided by the Pathology Department of Oncology Centre. The tumour tissues were analysed and histologically classified according to the World Health Organisation, graded using the Elston and Ellis grading system and grouped into stages according to TNM classification according to the American Joint Committee on Cancer (AJCC; 7th ed.). Intrinsic subtypes of breast cancer were established by applying immunohistochemical marker profiles. The study protocol and the key inclusion and exclusion criteria of patients who were eligible for the research are presented in Figure 1.

### 2.2. Treatment Plan and Therapeutic Procedures

All breast cancer subjects included in the study underwent surgery as a primary treatment, including mastectomy, breast-conserving surgery (BCS) or modified radical mastectomy (MRM). None of the patients had received any treatment before the surgical operation. The primary surgery was followed by postoperative adjuvant treatment, consisting of radiotherapy, brachytherapy, hormone therapy, chemotherapy or immunotherapy based on the tumour profile. The frequency of application of the particular therapies is presented in Table 1. Post-surgery radiotherapy was used as a standard therapeutic option in the postoperative setting mainly in patients after BCS. The radiation was delivered with 6/15 MV X-ray given in 17–20 fractions over 4–6 weeks. Total doses ranged from 42.5 to 50 gray (Gy). High dose rate (HDR) brachytherapy as a boost treatment with an internal beam to the tumour bed was administrated with a single fraction of 10 Gy. Twenty-seven women were treated with adjuvant anthracycline-based chemotherapy and four received non-anthracycline regimens according to standard protocols indicating from three to six cycles. Oestrogen-receptor (ER)-positive patients were submitted to endocrine therapy with tamoxifen (Egis Pharmaceuticals, Budapest, Hungary), aromatase inhibitors (AIs) (Arimidex (anastrozole), AstraZeneca, Cambridge, UK) or a combination of tamoxifen and AIs. One ER-positive patient did not receive endocrine therapy due to a small tumour diameter. The type of endocrine treatment depended on menopausal status. Almost all patients with HER2 amplification received targeted immunotherapy (Trastuzumab). All IBrC patients enrolled in this study completed their entire recommended treatment regimen.

### 2.3. Clinical Outcomes and Survival Data

The follow-up period was counted from the date of IBrC diagnosis to the last date of contact. The duration from study enrolment to the date of relapse was determined as the duration of disease-free survival (DFS), and the duration until the patient was last seen or had died was determined as the overall survival time (OS). During the median follow-up period of 71 months (interquartile range: 68.5–74.5 months), 11 patients presented with disease progression including two locoregional recurrences or distant metastases (recurrence rate 18.33%) and nine deaths (death rate 15%).

### 2.4. Laboratory Assays

#### 2.4.1. Blood Sampling

Blood samples for testing TF, TFPI, t-PA, PAI-1 antigen (concentrations) and TF, TFPI activities were collected from each enrolled patient twice according to standard procedures. The first blood specimen collection took place 24 h before the scheduled primary surgery. The second blood collection occurred 8 months after tumour removal surgery. During the period between the first and second blood collections, all patients underwent adjuvant anticancer therapy. Peripheral blood from patients was drawn into sterile cooled tubes (Becton Dickinson Vacutainer R System, Plymouth, UK) containing 0.13 mol/L trisodium citrate (final blood to anticoagulant ratio 9:1). The plasma was separated by centrifugation for 15 min at 3000× *g* at +4 °C and kept at −80 °C until assayed but for no longer than 6 months.

#### 2.4.2. Haemostatic Parameters

To evaluate the plasma haemostatic parameters concentrations, we used the enzyme-linked immunosorbent assay (ELISA). The ELISA analyses were performed using commercial ELISA kits. The concentrations of TFPI, TF and PAI-1 were defined using the IMUBIND^®^Tissue Factor, IMUBIND^®^TFPI and IMUBIND^®^Plasma PAI-1 ELISA test Kit 96-Well Plate Assay, Sekisui Diagnostics, LLC, Stamford, CT, USA, respectively. The concentration of t-PA was measured using the AssayMaxTM Human t-PA ELISA Kit, Assaypro LLC, St. Charles, MO, USA. The plasma activities of TF and TFPI were assayed with chromogenic assays, the ACTICHROME^®^TF and ACTICHROME^®^TFPI-tests (Sekisui Diagnostics, LLC, Stamford, CT, USA), respectively.

#### 2.4.3. Immunohistochemistry Staining

The ER and PR status, expression of human epidermal growth factor receptor 2 (HER2) and Ki67-proliferation index were measured in specimens of breast tumour tissue by immunohistochemistry methods. ER and PR status were quantitatively evaluated using the VENTANA BenchMark system (Ventana Medical Systems, Tucson, AZ, USA) with anti-ER clone SP1 and anti-PR clone 1E2 as the primary antibodies. Tumours were considered as positive for ER and PR if more than 1% of tumour nuclei were stained independently of staining intensity in accordance with the recommendations of the American Society of Clinical Oncology/College of American Pathologists. HER2 immunostaining was performed using VENTANA anti-HER2/neu (4B5) antibodies. The intensity of the HER2 expression was scored as HER2-negative = 0 or 1+ and HER2-positive = 3+. Tumours with scores of 2+ were taken as equivocal cases, which were further recommended for fluorescence in situ hybridisation (FISH) analysis. The Ki67 antigen staining was performed using the monoclonal mouse antibody (Auto-stainer Link 48, Agilent Technologies, Santa Clara, CA, USA). The Ki67 score was calculated as the percentage of immunostained cells. The optimal cut-off for a high versus low Ki67 score was defined as the 20% threshold.

### 2.5. Statistical and Bioinformatics Analysis

The collected data were analysed using Statistica v. 13.1 (StatSoft, Cracow, Poland). The normality distribution of the data set was tested by the Shapiro-Wilk test. Median and interquartile ranges (IQR) were calculated for descriptive non-parametric data. The Wilcoxon pair rank test was used to compare the observed differences in post-treatment haemostatic parameter levels with respect to pre-treatment values. The significance of differences in plasma haemostatic parameter levels between the subgroups was determined by the Mann–Whitney U test or ANOVA test. The receiver-operating characteristics (ROC) curves were plotted to evaluate the diagnostic accuracies of the studied haemostatic parameters for the prediction of DFS in IBrC patients. The diagnostic accuracy of each biomarker was expressed as the area under curve (AUC). The DFS and OS analyses were described by Kaplan–Meier curves and compared between groups with log-rank tests. The Kaplan–Meier curves were designed using MedCalc software. The hazard ratios (HR) for potential prognostic factors of IBrC recurrence were determined by univariate and multivariate Cox proportional hazard regression with 95% confidence intervals. The level of significance was considered as *p* < 0.05.

## 3. Results

### 3.1. Characteristics of Invasive Breast Cancer Patients

The basic demographic and descriptive characteristic of this study cohort are shown in Table 1. The median age of the patients was 56 years (IQR 51–59 years). At the time of diagnosis, 14 (23%) patients were premenopausal whereas 46 (77%) were postmenopausal. The median BMI was 25.0 kg/m^2^ (IQR 22.4–28.9 kg/m^2^). Half of the patients (50%) were in the BMI ≤ 24.9 kg/m^2^ group. The others were classified as overweight (BMI 25–29.9 kg/m^2^) or obese (BMI ≥ 30 kg/m^2^). Tumour sizes ranged from 0.4 to 3.5 cm, with a median size of 1.55 cm (IQR 1.2–2.3 cm). Regarding histological type, 85% of subjects had invasive ductal carcinoma and 15% had invasive lobular carcinoma. The majority of the analysed group were patients in stage IIA/IIB (61%) according to the TNM staging system. The remaining patients (39%) were in stage IA. The ER (+), PR (+) and HER2 (+) were determined for 85%, 75% and 13% of the cases, respectively. Based on molecular classification, the luminal A type was the most common subtype in this study (57% of the patients) whereas 26 (43%) patients had non-luminal A tumours, including luminal B, non-luminal HER2+ and triple-negative.

### 3.2. Clinical Presentation of Patients Regarding to Haemostatic Parameters

The detailed analysis of variabilities in the antigen (concentration) and activity of haemostatic parameters with respect to demographic and clinicopathological characteristics are presented in Tables S1 and S2 (see the Supplementary Materials for further details). It was found that most of the demographic features (age, menopausal status, BMI, parity status and smoking status), histopathological (histological grade and histological type) and clinical characteristics (TNM classification, molecular subtype and Ki67 expression) had no significant impact on the baseline and post-treatment TFPI, TF, t-PA, PAI-1 antigen levels and TFPI, TF activities. However, we noticed a tendency toward a higher pre-treatment TFPI activity in patients with tumour localised in the left breast; higher post-treatment TFPI activity in patients with grade G3, stage N1 and Ki67 ≥ 20%; higher pre-treatment TFPI antigen in patients ≥ 56 years with BMI ≥ 25 kg/m^2^; higher pre-treatment TF activity in patients with non-luminal A cancer subtype and Ki67 ≥ 20%; higher post-treatment t-PA antigen in patients with invasive ductal carcinoma; higher pre-treatment PAI-1 activity in patients with tumour localised in the left breast, invasive ductal carcinoma and post-treatment PAI-1 activity in patients with BMI ≥ 25 kg/m^2^ (*p* < 0.05).

### 3.3. The Impact of Adjuvant Treatment on the TFPI, TF, t-PA, PAI-1 Antigen Concentrations and TFPI, TF Activities

In the present study, we examined the potential impact of anticancer treatment on the TF, TFPI, t-PA, PAI-1 antigen concentrations and TF, TFPI activities in breast cancer patients. Baseline levels of the haemostatic parameters were examined according to the response to primary and adjuvant treatment. The primary surgery as well as adjuvant therapy significantly increased the plasma concentration of TF, the PAI-1 antigen and also activity of TF and TFPI but significantly decreased the t-PA antigen concentration, as shown in Table 2 and Table 3. However, we found no significant change in the TFPI antigen concentration before or after anticancer treatment.

Additionally, we compared the pre- and post-treatment concentrations and activities of the studied haemostatic parameters depending on the form of adjuvant treatment applied (Table 4 and Table 5). The results showed that treating with radiotherapy, chemotherapy or hormone therapy as the only form of adjuvant treatment (monotherapy) does not lead to statistically significant differences between the baseline and post-treatment haemostatic biomarker levels. The only exception is the PAI-1 antigen, in connection with which we noticed a significant increase in concentration in response to monotherapy. However, the implementation of combination therapy, which is a combination of various forms of adjuvant treatment, significantly affects changes in the concentrations and activities of almost all the investigated biomarkers in the general IBrC cohort. Both therapy combined with chemotherapy and therapy combined with hormone therapy significantly increased the concentrations of plasma TF and the PAI-1 antigen as well as activity of TFPI and TF but significantly decreased the t-PA antigen (*p* < 0.05). These results lead to the initial conclusion that combination adjuvant therapy appears to have a greater impact on the levels of haemostatic parameters than adjuvant therapy when applied as a monotherapy.

### 3.4. Predictive Value of Haemostatic Parameters for Clinical Outcome

**Figure 2:**
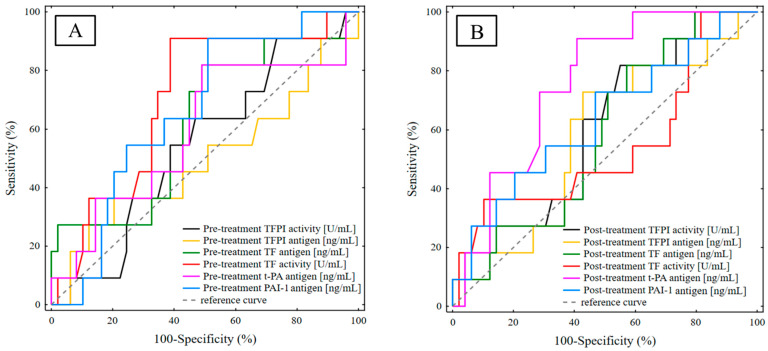
ROC curve analysis of the investigated haemostatic biomarkers: (**A**) ROC curve analysis of the pre-treatment haemostatic biomarkers; (**B**) ROC curve analysis of the post-treatment haemostatic biomarkers.

Receiver-operating characteristic (ROC) analysis was performed to examine diagnostic accuracies and confirm the usefulness of the studied haemostatic parameters for the prediction of the DFS (Figure 2). The AUC was estimated to summarise each biomarker’s classification accuracy across the cut-off values calculated based on the maximum value of the Youden index (Table 6 and Table 7). According to the results, the pre-treatment TF activity (AUC = 0.701, *p* = 0.0143) and PAI-1 (AUC = 0.659, *p* = 0.0472) concentration are considered to be the strongest predictors of disease relapse and may effectively predict breast cancer recurrence before the application of adjuvant therapy. The pre-treatment TF activity of 13.32 U/mL and pre-treatment PAI-1 concentration of 36.46 ng/mL were identified as the best cut-off values to discriminate relapsed from non-relapsed IBrC patients with a sensitivity of both 90.9% and specificity of 61.2%, 49.0%, respectively. Furthermore, comparison of individual post-treatment biomarkers revealed that after adjuvant treatment the t-PA antigen had the highest AUC value (AUC = 0.757, *p* = 0.0001) indicating that a t-PA antigen concentration of 3.13 ng/mL may reliably predict post-treatment outcomes in patients’ breast cancer with a specificity of 59.2% and a sensitivity of 90.9%. Despite the fact that the AUC^ROC^ for other studied haemostatic parameters were above 0.5, the *p*-values were >0.05, and thus the strong diagnostic potential for predicting future clinical outcome is not achieved.

### 3.5. Survival Analysis Regarding TFPI, TF, t-PA, PAI-1 Antigen Concentrations and TFPI, TF Activities

**Figure 3:**
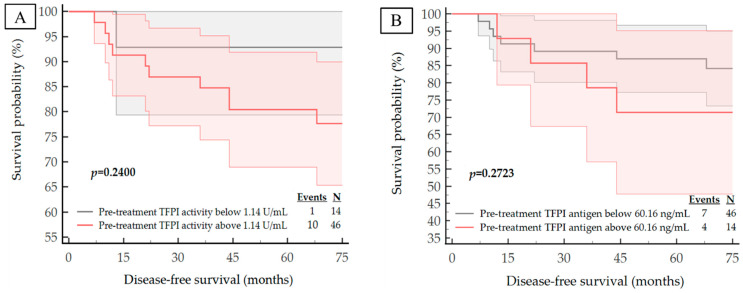

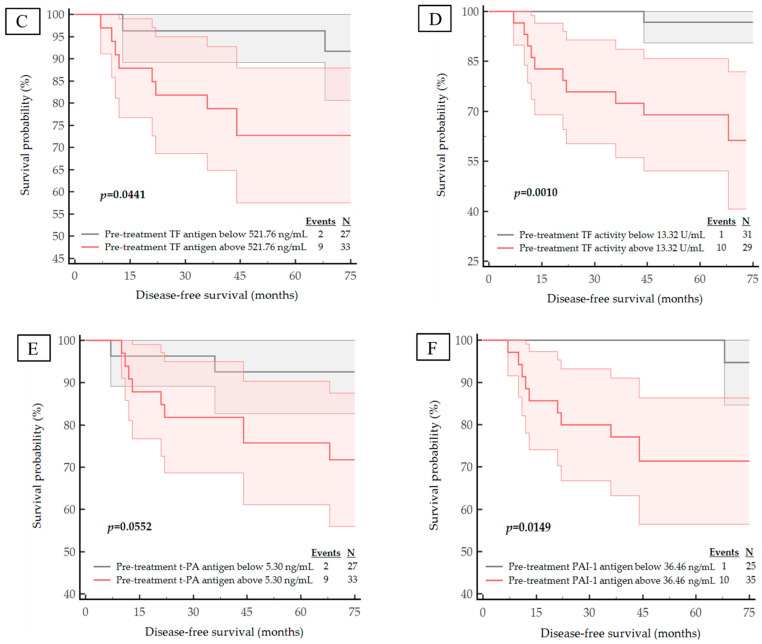
Kaplan–Meier survival plots for the DFS analysis of the studied population regarding to: (**A**) pre-treatment TFPI activity; (**B**) pre-treatment TFPI antigen; (**C**) pre-treatment TF antigen; (**D**) pre-treatment TF activity; (**E**) pre-treatment t-PA antigen; (**F**) pre-treatment PAI-1 antigen.

**Figure 4:**
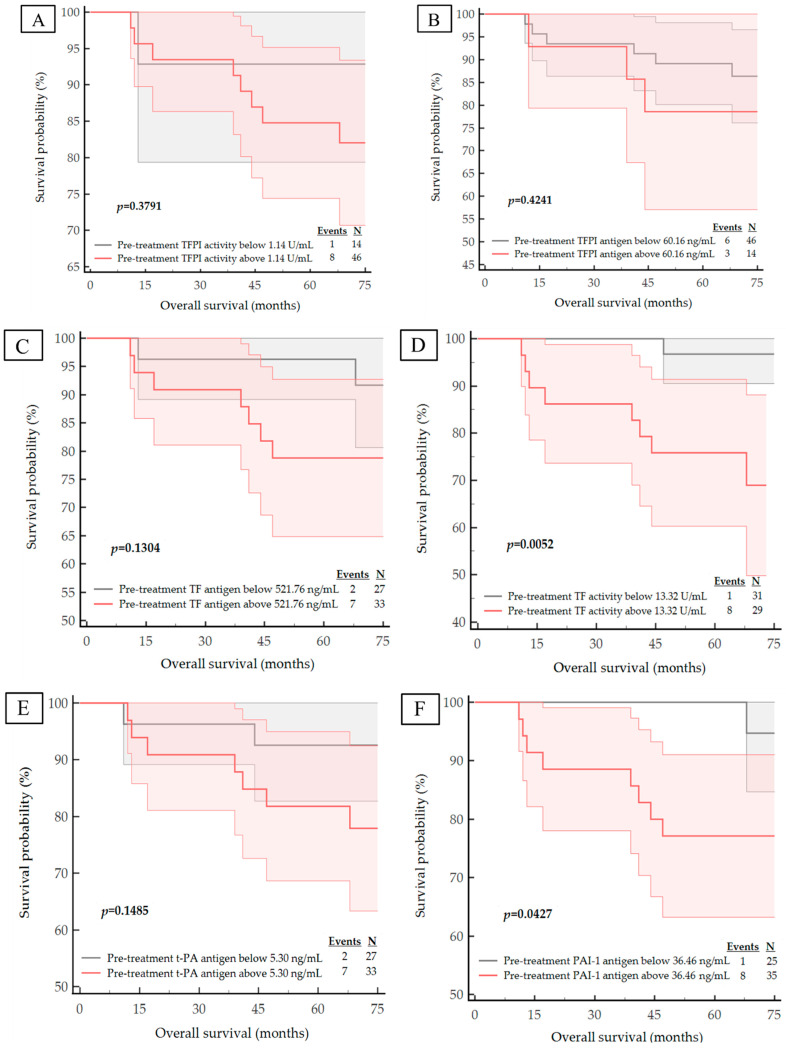
Kaplan–Meier survival plots for the OS analysis of the studied population regarding to: (**A**) pre-treatment TFPI activity; (**B**) pre-treatment TFPI antigen; (**C**) pre-treatment TF antigen; (**D**) pre-treatment TF activity; (**E**) pre-treatment t-PA antigen; (**F**) pre-treatment PAI-1 antigen.

**Figure 5:**
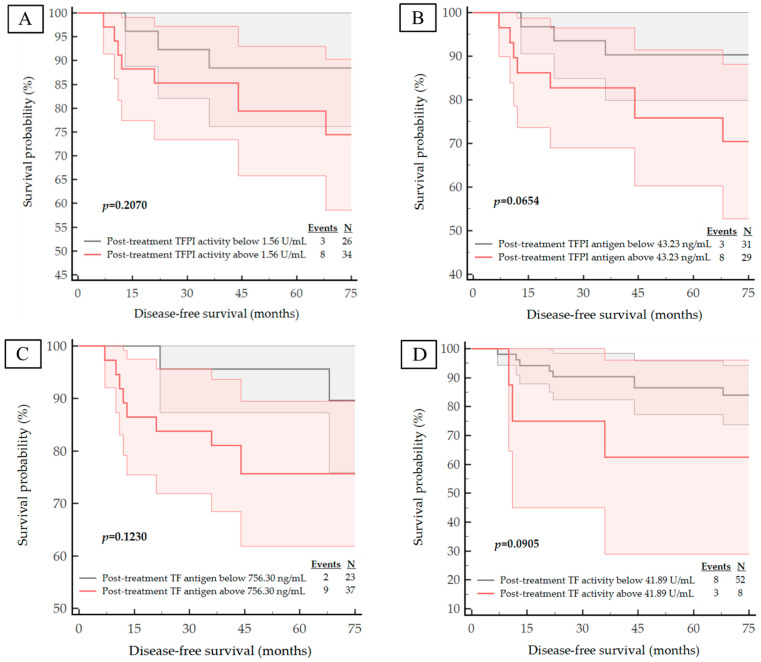

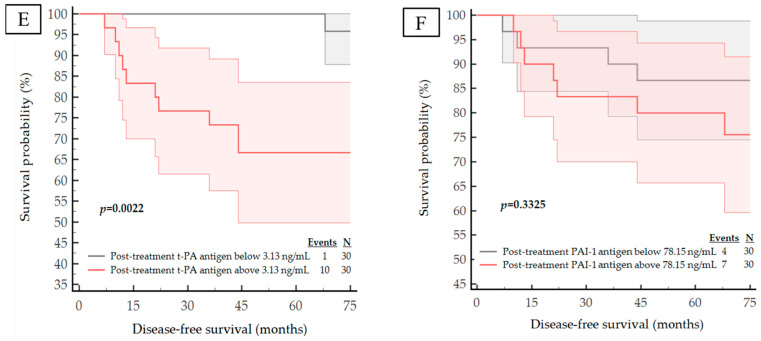
Kaplan–Meier survival plots for the DFS analysis of the studied population regarding to: (**A**) post-treatment TFPI activity; (**B**) post-treatment TFPI antigen; (**C**) post-treatment TF antigen; (**D**) post-treatment TF activity; (**E**) post-treatment t-PA antigen; (**F**) post-treatment PAI-1 antigen.

**Figure 6:**
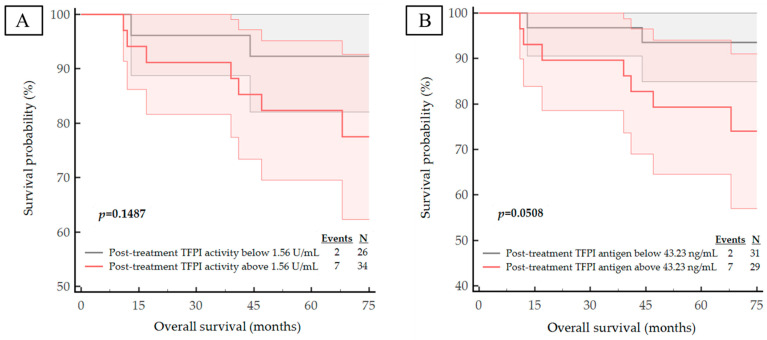

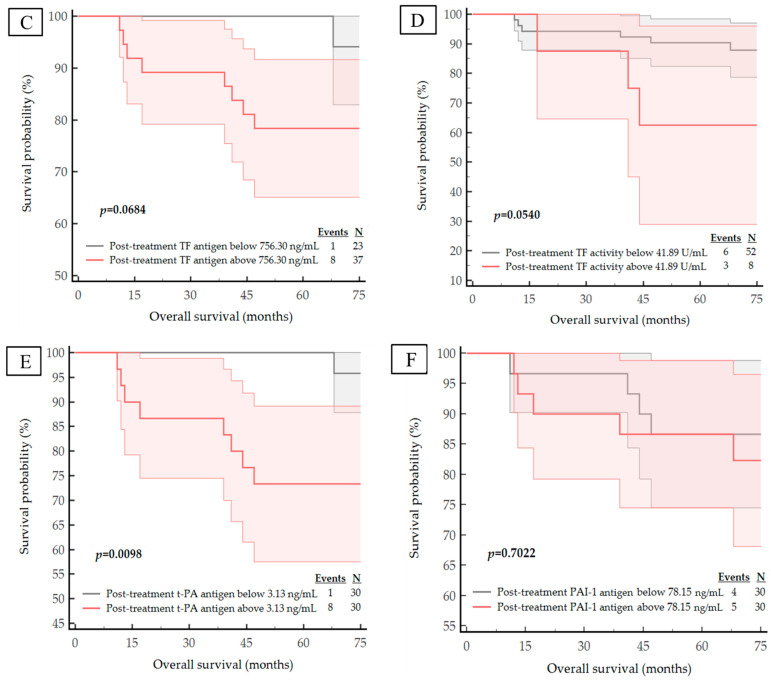
Kaplan–Meier survival plots for the OS analysis of the studied population regarding to: (**A**) post-treatment TFPI activity; (**B**) post-treatment TFPI antigen; (**C**) post-treatment TF antigen; (**D**) post-treatment TF activity; (**E**) post-treatment t-PA antigen; (**F**) post-treatment PAI-1 antigen.

We next examined the correlation of the studied haemostatic biomarker concentrations and activities with important clinical outcomes in breast cancer. During a median follow-up of 6 years, nine patients had died from systemic metastatic disease and 11 subjects presented with disease progression expressed by distant metastases. The median DFS and OS for the entire study cohort was 71 months (IQR 65–74 months). The corresponding Kaplan–Meier curves are shown in Figure 3, Figure 4, Figure 5 and Figure 6. The calculated ROC optimal cut-off values of the investigated haemostatic parameters were used as a cut-off point to divide patients into two groups: the group with baseline/post-treatment level below the cut-off point and the group with baseline/post-treatment level above the cut-off value.

The Kaplan–Meier curves for high versus low pre-treatment concentrations/activities of the haemostatic parameters demonstrated that high expression pre-treatment—TF antigen (*p* = 0.0441), TF activity (*p* = 0.0010) and PAI-1 antigen (*p* = 0.0149)—corresponded with a significantly worse DFS. Similarly, high levels of both pre-treatment TF activity (*p* = 0.0052) and PAI-1 antigen (*p* = 0.0427) are correlated with significantly shorter OS.

Examination of the Kaplan–Meier curves for post-treatment haemostatic biomarker levels revealed that patients with higher post-treatment concentration of the t-PA antigen also appeared to have a poorer prognosis for DFS and OS (*p* = 0.0022 for DFS; *p* = 0.0098 for OS), as shown in Figure 5E and Figure 6E. Although the *p*-value for the post-treatment TFPI antigen concentration with respect to OS was *p* = 0.0508, it was considered to be statistically significant due to its proximity to *p* < 0.05; thus, we postulate that patients with a high post-treatment TFPI concentration have a worse OS outcome.

Additionally, we performed a DFS and OS analysis regarding the form of adjuvant treatment applied. It was found that the type of adjuvant treatment did not significantly affect DFS and OS. An important limitation of this analysis is certainly the varied and unequal number of individual subgroups. While all women in the cohort underwent surgery (mastectomy, BCS or MRM) as a primary treatment, adjuvant treatment varied according to breast cancer subtype. The detailed analysis is presented in Figure S1 (see the Supplementary Materials for further details).

According to the Kaplan–Meier survival analysis we suggest that higher pre-treatment TF antigen, TF activity, PAI-1 antigen concentration and post-treatment t-PA and TFPI antigen concentration may act as a negative prognostic factor for disease recurrence and may increase the risk of death due to breast cancer.

### 3.6. Univariate and Multivariate Analyses of Haemostatic Parameters Contributing to DFS

The prognostic value of the haemostatic biomarkers was confirmed by univariate and multivariate Cox proportional hazards regression analyses (Table 8 and Table 9). We used univariable regression to identify each of studied parameters as a risk factor for cancer recurrence. Next, we established a multivariable Cox model to determine an independent predictive value of the investigated haemostatic markers after adjusting for BMI, age at the time of diagnosis, TNM staging, molecular subtype, histological type, nodal involvement and tumour diameter. The calculated HR is presented to quantify its impact on DFS.

The univariate analysis identified that high levels of both pre-treatment TF activity and PAI-1 antigen significantly contributed to shorter DFS. According to the calculated ROC optimal cut-off point values, pre-treatment TF activity > 13.32 U/mL and pre-treatment PAI-1 concentration > 36.46 ng/mL are positively correlated with breast cancer recurrence (HR = 14.33, 95% CI = 1.81–113.28, *p* = 0.0116; HR = 8.39, 95% Cl = 1.07–65.58, *p* = 0.0427, respectively). At the same time, the univariable analysis of post-treatment haemostatic biomarkers suggested that patients with a post-treatment t-PA concentration > 3.13 ng/mL appear to have a 11.29-times higher risk of disease-specific relapse (HR = 11.29, 95% CI = 1.43–89.25, *p* = 0.0216).

In a multivariate Cox analysis, by including other-related parameters in a model, we found no significant association between the risk of IBrC relapse and the studied variables (*p* > 0.05).

## 4. Discussion

Haemostasis is an essential process for maintaining the integrity of the circulatory system, and involves several mechanisms such as vasoconstriction, platelet activation, coagulation and fibrinolysis [17]. The association between malignancy and disturbances in the haemostasis balance has been studied for almost two centuries [18]. In recent years, a large number of researchers have reported a significant role of both coagulation and fibrinolytic parameters in the pathogenesis of several cancers as prognostic and predictive indicators of survival and future clinical outcomes [19]. There is much evidence indicating that components of the haemostatic system contribute to the fundamental aspects of tumour biology, such as angiogenesis, metastasis, cancer progression and modulation of immune responses [20,21]. Moreover, the haemostatic system is not only affected by malignancy, but primary and adjuvant cancer-related treatment may also play a crucial role in the dysregulation of haemostatic interactions [22].

In this study, as a first step, we investigated the prognostic role of haemostatic parameters,TFPI, TF, t-PA and PAI-1, as potential prognostic factors of breast cancer recurrence and OS. Furthermore, we decided to establish the potential influence of adjuvant treatment on the concentrations and activities of the investigated haemostatic biomarkers. This approach has facilitated the determination of the effect of anticancer treatment on survival with respect to coagulation and fibrinolysis components.

### 4.1. TF, TFPI, t-PA and PAI-1 as Prognostic Indicators of Survival

The correlation between TFPI, TF, t-PA and PAI-1 values and the probability of IBrC recurrence has been investigated by numerous researchers, and this observation is consistent with our study. Interestingly, the 71-month median follow-ups revealed a significantly higher incidence of cancer-specific death or disease relapse in IBrC patients with higher baseline levels of TF and the PAI-1 antigen. According to the post-treatment analysis, we also found that higher concentrations of t-PA and TFPI strongly correspond with a worse survival outcome. Using a univariate Cox analysis, we confirmed the predictive value of the haemostatic biomarkers as indicators of DFS.

Patients in the group with baseline TF activity > 13.32 U/mL presented an over 14-times higher risk of disease relapse (HR = 14.33; 95% Cl = 1.81–113.28) and obtained a significantly shorter DFS and OS. Our findings corroborate the results of research conducted between 1983 and 1996 by Ueno et al. who performed an ELISA in the plasma of 67 breast cancer patients and immunohistochemistry in 213 breast cancer tissues. The authors revealed that TF overexpression is associated with up-regulated TF plasma levels and poor OS in primary and recurrent breast cancer patients. They suggested that TF promotes cancer invasion and metastasis both through hypercoagulation initiation and through activation of the intracellular signalling pathways in TF-expressing cells [23]. Surprisingly, Stämpfli et al. obtained results completely inconsistent with our current study. Although, the authors reported that TF was expressed in 99% of breast cancer specimens, they did not support a prognostic impact of TF expression on a breast cancer patient’s OS [24]. However, when evaluating this discrepancy, it must be taken into account that the above-mentioned study used a cancer tissue as a biological material, while our study assessed TF activity in plasma.

Furthermore, a pre-treatment PAI-1 concentration > 36.46 ng/mL also corresponds with a poorer outcome (shorter DFS and OS) and shows an association with an over 8-times-higher risk of cancer recurrence. As a natural inhibitor of urokinase-type plasminogen activator (u-PA) and tissue-type plasminogen activator (t-PA)—two molecules commonly associated with angiogenesis, metastasis and cancer progression—it was expected that PAI-1 would successfully prevent the development of cancers. In contrast, many studies have revealed that high levels of PAI-1 are correlated with a poor rather than a favourable clinical outcome [25,26]. The inverse relation between PAI-1 level and survival has been confirmed by Ferroni et al.’s research. Using Kaplan–Meier analysis and a Cox proportional hazard model, the authors reported that an elevated plasma PAI-1 level had a negative prognostic impact in terms of both relapse-free (RFS) and OS and serves as an independent biomarker for predicting the disease outcome in breast cancer patients [27]. The potential paradoxical pro-tumourigenic mechanism of PAI-1 in cancer pathogenesis may result from its anti-apoptotic, anti-protease and vitronectin-binding functions leading to concentration-dependent pro-angiogenic activity [26,28]. It has been proved that PAI-1 stimulates angiogenesis by initiating the migration of endothelial cells from vitronectin towards fibronectin, secondarily promoting the elongation of micro-vessels supported by fibronectin [29].

With respect to post-treatment analysis, we postulate that a t-PA concentration > 3.13 ng/mL may predict a poor clinical outcome in terms of DFS and OS. Our analysis showed that most patients with cancer relapse or cancer-specific death displayed elevated plasma levels of post-treatment t-PA. A negative association between the post-treatment t-PA concentration and survival was finally confirmed using a Cox proportional hazards regression analysis which showed that patients with higher after-therapy t-PA levels have an 11.29-times increased risk of disease recurrence (HR = 11.29, 95% CI = 1.43–89.25). There is not much data supporting a potential predictive value of t-PA with respect to the adjuvant chemotherapy received. Teliga-Czajkowska et al. presented that a high t-PA plasma level at the onset of chemotherapy was associated with shorter OS and DFS in patients with ovarian cancer, but they found no significant differences in DFS and OS after three and six cycles of chemotherapy [30]. Another study conducted by Corte et al. showed no significant relation between intra-tumoural t-PA levels and RFS and OS prognosis in breast cancer patients in accordance with the type of systemic adjuvant therapy received. The authors controvert the clinical prognostic usefulness of t-PA and its predictive value in systemic adjuvant therapy [31]. Further research is needed to establish these inconsistent results.

Similarly, we observed a negative impact of post-treatment TFPI concentration in terms of OS. Unfortunately, we did not find any study which investigated the prognostic value of post-treatment TFPI in breast or other types of cancer.

### 4.2. The Impact of Adjuvant Therapy on Haemostatic Parameter Levels

Our primary goal was to determinate the potential impact of adjuvant treatment on the TFPI, TF, t-PA and PAI-1 levels to better understand the effect of anticancer therapy on coagulation and the fibrinolysis process. We came to the conclusion that both therapy combined with chemotherapy and therapy combined with hormonotherapy, as well as radiotherapy, significantly increased the concentrations of plasma TF and the PAI-1 and also activity of TF and TFPI, but significantly decreased the t-PA antigen (*p* < 0.05). What is more, the use of monotherapy has no significant effect on haemostatic biomarker levels. Based on these results, we postulate that TFPI, TF, t-PA and PAI-1 may act as biomarkers for monitoring therapy in patients with breast cancer. As is known, the main purpose of systemic adjuvant treatment is to improve the cure rates by reducing and controlling distant and local recurrence [32,33]. Despite the general positive effect of adjuvant treatment on decreasing the risk of breast cancer recurrence and mortality, there is clear available evidence that post-surgery hormonal therapy and cytotoxic chemotherapy contribute to the unfavourable hypercoagulable state [22].

Since we have revealed that elevated levels of TFPI, TF, t-PA and PAI-1 are associated with a poor prognosis in IBrC patients, we propose that adjuvant therapy negatively affects haemostasis by promoting a hypercoagulable state along with initiating imbalance in the fibrinolysis process. Khorana et al. reported elevated levels of plasma TF antigen and activity during the course of chemotherapy and suggested its predictive role for subsequent venous thromboembolism (VTE) events in patients with pancreatic cancer [34]. In addition, Bertomeu et al. demonstrated that chemotherapeutic drugs administrated to stage II breast cancer patients may induce profound changes at the endothelial cell level and increase levels of procoagulant molecules, especially cytokines, which subsequently affect the thrombotic process [35]. Based on this observation, we speculate that direct post-chemotherapy damage to the vascular endothelium, which is the mean surface of TF exposition, can lead to increased expression and activity of TF, promoting the hypercoagulable state. With regard to the fibrinolytic factors, Rella et al. observed a significant increase in plasma PAI-1 antigen levels after starting chemotherapy, lasting until the last cycle, which is also in line with our findings [36]. Similar to the above, it may be hypothesised that chemotherapy-induced endothelial injury enhances the production and secretion of PAI-1, the most important physiological inhibitor of fibrinolysis, and results in an antifibrinolytic condition that may contribute to the pathogenesis of thrombotic microangiopathy [37]. Furthermore, PAI-1, as a serpin-inhibiting caspase-3, promotes cell survival and protects tumour cells from chemotherapy-induced apoptosis [26]. Moreover, endocrine therapy with tamoxifen is an additional risk factor for VTE in breast cancer patients. Interestingly, Saphner et al. noted that the combination of chemotherapy and tamoxifen was associated with higher risk of venous and arterial thromboembolic complications than chemotherapy alone in premenopausal breast cancer patients [38]. Although there are plenty of studies demonstrating that tamoxifen activity might elevate the risk of VTE through the depletion of antithrombin, protein C and protein S, there are few studies assessing the effects of hormone treatment on the haemostatic biomarkers investigated in our study [39,40,41,42]. However, Trappenburg et al. demonstrated that endocrine therapy increases the number of circulating TF-bearing microparticles and heightens the procoagulant state in breast cancer patients [43]. Unfortunately, the mechanism by which endocrine therapy contributes to the increase in TF in the course of VTE is not clearly understood. According to the impact of adjuvant therapy on TFPI, we found completely inconsistent results to our current study in the available literature. Aharon et al. observed a significant decrease in the levels of TFPI-bearing extracellular vesicles at the end of neo-adjuvant and adjuvant chemotherapy in breast cancer treatment [44]. Likewise, a study conducted by Blondon et al. demonstrated a significant association of tamoxifen use with decreases in plasma levels of TFPI, which may be an important explanation for the procoagulant risk of tamoxifen in breast cancer patients [45]. Therefore, based on our current results, we hypothesise that elevated post-treatment plasma activity of TFPI results in the compensatory release of TFPI associated with up-regulation of the expression of TF both by tumour cells and endothelial cells damaged by adjuvant therapy. Finally, our findings concerning post-treatment changes in t-PA levels were consistent with the study conducted by Al-Youzbaki et al. where a significant decrease in the t-PA serum level in breast cancer patients who received six cycles of chemotherapy was observed [46]. Moreover, Lox et al. observed a significant increase in t-PA and PAI-1 levels in 26 tamoxifen-treated breast cancer patients, which is also in line with our findings [47]. Since we have proved a negative association between the post-treatment t-PA concentration and survival, we propose that t-PA may act as biomarker for monitoring therapy in breast cancer patients.

### 4.3. Limitations of the Study

There are certain limitations to this study, consisting of its small sample size and the lack of a control group, that may have implications for future research. Nevertheless, the study was performed in a daily clinical routine, and the sample size was dependent on receiving patients’ consent for participation. Furthermore, very restrictive inclusion and exclusion criteria also influenced the limited number of patients in the project. The present study excluded the patients who have undergone neoadjuvant therapy, what would disturb the assessment of the condition of the vascular endothelium and the analysed haemostatic factors. Nowadays, the standard treatment of patients in stage III or higher requires treatment with neoadjuvant chemotherapy, followed by surgery. Thus, we enrolled to the study patients only in I and II stage of invasive breast cancer. It is worth noting that our cohort was from a single institution, and thus the results of our research should be further validated in a multi-centre study recruiting a larger population and including a control group. Despite some limitations, the main conclusions of this investigation are consistent with recent trial and population studies performed by other authors. Thus, the strength of our research is expressed by the use of samples collected and processed using standard operating procedures. Additionally, in late-stage cancer patients, numerous factors associated with cancer status might affect the haemostatic parameters. Hence, the elimination of patients with late-stage BrC allowed us to investigate specifically the association between stage IA–IIB of BrC and haemostatic profile, regardless of the essential confounders.

## 5. Conclusions

Our results point out that adjuvant therapy significantly increased the concentrations of plasma TF and the PAI-1 as well as activity of TF and TFPI but significantly decreased the levels of the t-PA antigen. Considering the role of haemostatic biomarkers and an enhanced effect of anticancer treatment on the hypercoagulable and hypofibrinolytic state, we suggest that breast cancer patients receiving adjuvant therapy have a higher risk of developing venous and arterial thromboembolic complications. Additionally, both pre-treatment TF activity and PAI-1 concentration and also post-treatment t-PA concentration were associated with the future outcomes of IBrC patients, since a pre-treatment TF activity above 13.32 U/mL, a pre-treatment PAI-1 concentration > 36.46 ng/mL and a post-treatment t-PA concentration > 3.13 ng/mL have been shown to promote the probability of recurrence and morbi-mortality in the IBrC cohort.

## Figures and Tables

**Table 1:** Presentation of IBrC patients according to demographic and clinical characteristics.

Demographic and Clinical Variables	Overall (n = 60; 100%)
Age	
<56 years	27 (45%)
≥56 years	33 (55%)
Menopausal status	
pre-menopause	14 (23%)
post-menopause	46 (77%)
Body mass index	
Normal ≤ 24.99 kg/m^2^	30 (50%)
Overweight or obese ≥ 25 kg/m^2^	30 (50%)
Parity status	
0	6 (10%)
1–2	41 (68%)
3 and more	13 (22%)
Smoking status	
Smokers	17 (28%)
Non-smokers	43 (72%)
Tumour localization	
Left breast	31 (52%)
Right breast	29 (48%)
Histological type	
IDC	51 (85%)
ILC	9 (15%)
Histological grade	
G1 + G2	47 (78%)
G3	13 (22%)
cT category (7th ed.)	
T1	37 (59%)
T2	23 (41%)
cN category (7th ed.)	
N0	42 (68%)
N1	18 (32%)
cStage (7th ed.)	
IA	24 (39%)
IIA + IIB	36 (61%)
Molecular subtype	
Luminal A	34 (57%)
Non-luminal A	26 (43%)
Molecular components	
ER (+)	51 (85%)
ER (−)	9 (15%)
PR (+)	45 (75%)
PR (−)	15 (25%)
HER2 (+)	8 (13%)
HER2 (−)	52 (87%)
Proliferation marker expression	
Ki67 < 20%	39 (65%)
Ki67 ≥ 20%	21 (35%)
Surgery type	
Mastectomy	5 (8%)
BCS	46 (77%)
MRM	9 (15%)
Radiotherapy	
Yes	49 (82%)
No	11 (18%)
Brachytherapy	
Yes	28 (47%)
No	32 (53%)
Chemotherapy	
Anthracykline	27 (45%)
Non-anthracycline	4 (7%)
No	29 (48%)
Endocrine therapy	
Tamoxifen	30 (50%)
Inhibitor aromatase	13 (22%)
Tamoxifen + inhibitor aromatase	7 (11%)
No	10 (17%)
Immunotherapy	
Yes	6 (10%)
No	54 (90%)

IDC: Invasive ductal carcinoma; ILC: Invasive lobular carcinoma; G1: low grade; G2: intermediate grade; G3: high grade; T1: tumour diameter ≤ 2 cm; T2: tumour diameter > 2 cm to ≤5 cm; N0: lack of lymph node metastases; N1: spread to axillary lymph nodes; ER: oestrogen receptor; PR: progesterone receptor; HER2: human epidermal growth factor receptor 2; Ki67: proliferation marker; BCS: breast-conserving surgery; MRM: modified radical mastectomy.

**Table 2:** TFPI activity, TFPI and TF antigen concentrations according to the type of surgery and adjuvant therapy in IBrC subjects.

Type of Treatment	Pre-Treatment TFPI Activity [U/mL]	Post-Treatment TFPI Activity [U/mL]	*p*-Value *	Pre-Treatment TFPI Antigen [ng/mL]	Post-Treatment TFPI Antigen [ng/mL]	*p*-Value **	Pre-Treatment TF Antigen [ng/mL]	Post-Treatment TF Antigen [ng/mL]	*p*-Value ***
Surgery type									
	1.28	1.68		61.88	32.87		466.34	822.71	
Mastectomy	1.26/1.36	1.56/1.96	0.2249	58.80/64.56	31.94/46.69	0.0796	392.64/577.40	452.43/841.96	0.2249
	1.29	1.64		43.74	42.57		552.70	834.90	
BCS	1.12/1.48	1.10/2.06	**0.0083**	37.12/50.40	38.13/52.48	0.9956	400.86/718.70	660.92/999.12	**<0.0001**
	1.40	1.96		40.68	46.53		592.28	789.20	
MRM	1.30/1.58	1.72/2.52	**0.0284**	37.64/55.00	43.23/98.36	0.3743	521.76/896.86	689.43/904.71	0.0506
Radiotherapy									
	1.28	1.70		43.44	42.77		562.47	831.91	
Yes	1.12/1.48	1.10/2.06	**0.0030**	37.64/50.80	38.39/51.59	0.8384	400.94/724.55	660.92/999.12	**<0.0001**
	1.40	1.72		53.92	44.84		521.76	822.71	
No	1.26/1.58	1.48/2.82	0.0619	39.08/61.88	31.94/98.36	0.5937	442.73/581.87	655.33/904.71	**0.0044**
Brachytherapy									
	1.31	1.54		42.68	42.69		537.87	803.05	
Yes	1.12/1.53	1.00/1.92	0.0776	35.82/48.94	38.47/53.11	0.3995	386.85/723.82	666.14/979.33	**0.0002**
	1.31	1.77		47.62	43.08		557.20	827.41	
No	1.15/1.49	1.39/2.20	**0.0015**	39.88/61.12	34.54/50.30	0.4001	439.56/689.41	658.13/1010.72	**<0.0001**
Chemotherapy									
	1.28	1.82		42.36	43.23		565.46	837.69	
Anthracykline	1.10/1.46	1.40/2.20	**0.0004**	34.36/61.88	37.87/53.46	0.6139	400.86/726.12	691.40/973.34	**0.0001**
	1.41	2.45		40.04	78.01		517.51	727.67	
Non-anthracycline	1.25/1.51	1.57/2.67	0.1441	36.24/48.00	53.33/104.68	0.1441	413.55/792.09	567.50/1049.22	0.0679
	1.30	1.50		46.44	41.91		520.76	789.20	
No	1.16/1.56	0.76/1.80	0.2993	40.68/53.92	33.76/46.51	0.2137	402.49/686.50	642.06/1002.68	**0.0001**
Endocrine therapy									
	1.30	1.48		45.46	42.22		572.93	774.18	
Tamoxifen	1.14/1.50	0.90/1.82	0.2059	36.04/55.00	33.01/47.86	0.1915	387.68/718.70	642.06/884.08	**0.0002**
	1.26	1.76		44.16	46.99		563.96	890.45	
Inhibitor aromatase	1.18/1.32	1.52/2.70	**0.0088**	40.68/60.36	42.77/105.84	0.1520	466.34/728.94	691.40/973.34	**0.0046**
	1.48	1.96		55.76	46.51		562.47	1022.32	
Tamoxifen+inhibitor aromatase	0.94.1.68	1.74/2.62	0.2367	44.68/63.04	35.44/52.48	0.3980	520.76/774.32	985.32/1267.00	**0.0180**
	1.38	1.84		40.50	40.29		439.56	725.55	
No	0.78/1.56	1.10/2.20	0.0745	32.68/43.44	37.87/52.76	0.3329	376.02/474.14	543.86/837.69	**0.0284**
Immunotherapy									
	1.42	2.17		42.22	55.21		481.71	696.06	
Yes	1.42/1.60	0.76/2.96	0.2489	34.36/58.80	42.61/59.47	0.0277	386.02/718.70	479.66/1002.42	0.0747
	1.29	1.69		44.58	42.65		552.70	827.31	
No	1.12/1.48	1.18/2.06	**0.0007**	38.00/55.76	35.44/49.00	0.3502	431.59/724.55	671.43/985.32	**<0.0001**

TFPI: Tissue factor pathway inhibitor; TF: Tissue factor; BCS: breast-conserving surgery; MRM: modified radical mastectomy. Data are expressed as median (Me) and the interquartile range (IQR) [lower quartile (Q1)/upper quartile (Q3)]; *p*-value * for differences between pre-treatment and post-treatment TFPI activity; *p*-value ** for differences between pre-treatment and post-treatment TFPI antigen; *p*-value *** for differences between pre-treatment and post-treatment TF antigen; significant differences are denoted by bold *p*-values.

**Table 3:** TF activity, t-PA and PAI-1 antigen concentration according to the type of surgery and adjuvant therapy in IBrC subjects.

Type of Treatment	Pre-Treatment TF Activity [U/mL]	Post-Treatment TF Activity [U/mL]	*p*-Value *	Pre-Treatment t-PA Antigen [ng/mL]	Post-Treatment t-PA Antigen [ng/mL]	*p*-Value **	Pre-Treatment PAI-1 Antigen [ng/mL]	Post-Treatment PAI-1 Antigen [ng/mL]	*p*-Value ***
Surgery type									
	13.70	41.37		5.10	2.30		38.52	78.46	
Mastectomy	10.61/23.59	12.12/42.63	0.2249	4.55/5.87	2.02/3.01	0.0796	36.46/58.30	45.47/81.25	0.0796
	12.46	23.72		5.70	3.03		37.38	78.68	
BCS	11.17/23.59	11.22/33.11	**0.0008**	4.28/6.87	2.05/4.96	**0.0001**	27.73/44.97	47.70/85.97	**<0.0001**
	29.88	32.97		6.86	3.28		40.80	73.27	
MRM	11.65/41.82	13.32/45.39	0.3743	4.14/7.78	1.98/3.95	0.0858	35.50/45.48	60.72/84.65	0.1097
Radiotherapy									
	12.46	20.30		5.69	2.93		37.90	79.31	
Yes	11.03/23.59	11.20/33.11	**0.0006**	4.30/7.12	2.02/4.55	**<0.0001**	27.87/45.48	47.70/85.97	**<0.0001**
	23.59	34.09		5.87	3.28		37.19	73.27	
No	11.65/39.18	123.32/43.56	0.2860	3.92/7.16	1.36/6.99	0.0754	35.50/44.60	47.83/79.44	0.0912
Brachytherapy									
	12.39	27.84		5.83	3.67		36.16	79.85	
Yes	11.28/26.40	14.41/33.28	**0.0067**	4.50/7.43	2.25/5.06	**0.0083**	27.52/41.82	48.40/87.42	**<0.0001**
	13.65	18.28		5.41	2.97		39.50	75.87	
No	10.50/27.03	11.24/39.95	**0.0215**	4.04/7.00	1.63/3.82	**0.0001**	31.41/49.71	47.02/81.41	**0.0004**
Chemotherapy									
	13.88	27.69		5.87	2.69		37.20	78.15	
Anthracykline	11.77/29.88	12.12/33.45	0.0517	4.34/6.87	1.79/4.21	**0.0006**	27.73/45.48	46.21/84.33	**<0.0001**
	27.14	22.74		5.19	2.84		39.35	73.93	
Non-anthracycline	7.89/46.00	13.05/41.14	0.7150	3.20/6.06	1.57/7.91	0.7150	34.10/126.33	58.95/82.05	0.7150
	11.80	27.13		5.69	3.14		37.55	79.31	
No	10.39/23.31	11.28/37.68	**0.0021**	3.99/7.72	2.30/5.02	**0.0008**	28.10/44.97	47.70/86.75	**0.0001**
Endocrine therapy									
	11.71	23.72		5.59	3.10		35.98	55.30	
Tamoxifen	10.61/23.59	12.12/38.10	**0.0010**	4.42/6.70	2.02/4.55	**0.0007**	28.10/44.60	44.20/81.25	**0.0002**
	23.31	17.10		6.44	3.14		37.55	84.18	
Inhibitor aromatase	12.31/29.59	11.28/33.45	0.4631	4.83/7.72	2.57/6.42	**0.0024**	35.12/45.30	79.20/90.08	**0.0277**
	13.70	27.99		4.30	6.84		49.60	85.97	
Tamoxifen+inhibitor aromatase	10.03/23.59	17.91/41.89	**0.0180**	3.86/6.06	1.18/11.20	0.6121	27.30/56.19	81.15/88.22	**0.0180**
	13.89	26.43		5.51	2.12		39.97	73.64	
No	12.31/24.17	10.04/30.19	0.7989	4.4.23/7.38	1.16/3.34	**0.0125**	30.27/44.46	68.42/82.66	**0.0069**
Immunotherapy									
	12.61	17.01		5.66	2.35		41.12	80.35	
Yes	11.77/15.20	15.65/20.30	**0.0006**	5.10/6.70	1.79/6.84	0.4631	37.90/49.60	49.48/81.43	**0.0277**
	13.24	27.84		5.70	3.14		37.20	78.03	
No	11.03/26.92	11.28/37.68	0.3454	4.23/7.16	2.05/4.94	**<0.0001**	28.10/45.30	47.33/85.97	**<0.0001**

TF: Tissue factor; t-PA: Tissue plasminogen activator; PAI-1: Plasminogen activator inhibitor-1; Data are expressed as median (Me) and the interquartile range (IQR) [lower quartile (Q1)/upper quartile (Q3)]; *p*-value * for differences between pre-treatment and post-treatment TF activity; *p*-value ** for differences between pre-treatment and post-treatment t-PA antigen; *p*-value *** for differences between pre-treatment and post-treatment PAI-1 antigen; significant differences are denoted by bold *p*-values.

**Table 4:** TFPI activity, TFPI and TF antigen concentrations according to the type of adjuvant therapy in IBrC patients.

Type of Adjuvant Therapy	Pre-Treatment TFPI Activity [U/mL]	Post-Treatment TFPI Activity [U/mL]	*p*-Value *	Pre-Treatment TFPI Antigen [ng/mL]	Post-Treatment TFPI Antigen [ng/mL]	*p*-Value **	Pre-Treatment TF Antigen [ng/mL]	Post-Treatment TF Antigen [ng/mL]	*p*-Value ***
Adjuvant therapy (all types)	1.31	1.71	**0.0004**	44.32	42.85	0.8195	552.70	827.31	**<0.0001**
	1.14/1.49	1.15/2.09		37.82/57.28	36.97/52.62		401.72/721.63	660.89/992.22	
Monotherapy	1.48	1.48	1.0000	53.92	33.71	1.0000	466.34	655.33	0.8434
	1.30/1.60	0.76/2.82		33.40/60.36	32.87/98.36		442.73/752.35	498.44/841.96	
Combination therapy with chemotherapy	1.30	1.87	**0.0003**	41.68	46.52	0.3820	566.96	834.90	**<0.0001**
	1.10/1.42	1.40/2.20		35.60/58.80	38.93/56.46		400.86/726.12	691.40/973.34	
Combination therapy with endocrine therapy	1.28	1.72	**0.0015**	44.48	43.23	0.8434	577.40	866.51	**<0.0001**
	1.14/1.48	1.30/2.08		38.00/55.76	38.39/52.48		431.59/726.12	679.30/1022.32	

TFPI: Tissue factor pathway inhibitor; TF: Tissue factor; BCS: breast-conserving surgery; MRM: modified radical mastectomy. Data are expressed as median (Me) and the interquartile range (IQR) [lower quartile (Q1)/upper quartile (Q3)]; *p*-value * for differences between pre-treatment and post-treatment TFPI activity; *p*-value ** for differences between pre-treatment and post-treatment TFPI antigen; *p*-value *** for differences between pre-treatment and post-treatment TF antigen; significant differences are denoted by bold *p*-values.

**Table 5:** TF activity, t-PA and PAI-1 antigen concentrations according to the type of adjuvant therapy in IBrC patients.

Type of Adjuvant Therapy	Pre-Treatment TF Activity [U/mL]	Post-Treatment TF Activity [U/mL]	*p*-Value *	Pre-Treatment t-PA Antigen [ng/mL]	Post-Treatment t-PA Antigen [ng/mL]	*p*-Value **	Pre-Treatment PAI-1 Antigen [ng/mL]	Post-Treatment PAI-1 Antigen [ng/mL]	*p*-Value ***
Adjuvant therapy (all types)	12.96	27.13	**0.0003**	5.70	3.07	**<0.0001**	37.73	78.31	**<0.0001**
	11.10/26.40	11.67/35.89		4.26/7.14	2.00/4.95		29.19/45.39	47.77/85.19	
Monotherapy	12.31	25.17	0.4990	7.16	3.01	0.1282	38.52	68.42	**0.0425**
	10.61/39.18	11.28/43.56		3.04/9.68	2.18/7.41		35.50/44.60	47.33/81.39	
Combination therapy with chemotherapy	13.79	27.41	**0.0270**	5.79	2.91	**0.0013**	37.55	78.31	**0.0001**
	11.77/29.88	12.12/33.45		4.42/6.86	1.98/4.21		30.27/45.48	47.83/84.33	
Combination therapy with endocrine therapy	12.46	27.13	**0.0002**	5.69	3.14	**0.0001**	36.50	79.20	**<0.0001**
	11.03/26.92	12.12/37.68		4.28/6.70	2.02/5.02		27.87/45.48	47.70/85.97	

TF: Tissue factor; t-PA: Tissue plasminogen activator; PAI-1: Plasminogen activator inhibitor-1; Data are expressed as median (Me) and the interquartile range (IQR) [lower quartile (Q1)/upper quartile (Q3)]; *p*-value * for differences between pre-treatment and post-treatment TF activity; *p*-value ** for differences between pre-treatment and post-treatment t-PA antigen; *p*-value *** for differences between pre-treatment and post-treatment PAI-1 antigen; significant differences are denoted by bold *p*-values.

**Table 6:** Results of diagnostic accuracy for individual pre-treatment haemostatic parameters.

ROC Data	Pre-Treatment TFPI Activity	Pre-Treatment TFPI Antigen	Pre-Treatment TF Antigen	Pre-Treatment TF Activity	Pre-Treatment t-PA Antigen	Pre-Treatment PAI-1 Antigen
AUC	0.545	0.496	0.620	0.701	0.596	0.659
Youden index	0.17	0.16	0.33	0.52	0.33	0.40
Cut-off point	1.14	60.16	521.76	13.32	5.30	36.46
Sensitivity (%)	90.9	36.4	81.8	90.9	81.8	90.9
Specificity (%)	26.5	79.6	51.0	61.2	51.0	49.0
Positive predictive value (%)	21.7	28.6	27.3	34.5	27.3	28.6
Negative predictive value (%)	92.9	84.8	92.6	96.8	92.6	96.0
Accuracy (%)	38.3	71.7	56.7	66.7	56.7	56.7
*p*-value	0.6224	0.9724	0.2088	**0.0143**	0.3449	**0.0472**

TFPI: Tissue factor pathway inhibitor; TF: Tissue factor; TF: Tissue factor; t-PA: Tissue plasminogen activator; PAI-1: Plasminogen activator inhibitor-1; significant differences are denoted by bold *p*-values.

**Table 7:** Results of diagnostic accuracy for individual post-treatment haemostatic parameters.

ROC Data	Post-Treatment TFPI Activity	Post-Treatment TFPI Antigen	Post-Treatment TF Antigen	Post-Treatment TF Activity	Post-Treatment t-PA Antigen	Post-Treatment PAI-1 Antigen
AUC	0.606	0.573	0.579	0.544	0.757	0.635
Youden index	0.27	0.30	0.25	0.26	0.50	0.26
Cut-off point	1.56	43.23	756.30	41.89	3.13	78.15
Sensitivity (%)	81.8	72.7	81.8	36.4	90.9	72.7
Specificity (%)	44.9	57.1	42.9	89.8	59.2	53.1
Positive predictive value (%)	25.0	27.6	24.3	44.4	33.3	25.8
Negative predictive value (%)	91.7	90.3	91.3	86.3	96.7	89.7
Accuracy (%)	51.7	60.0	50.0	80.0	65.0	56.7
*p*-value	0.2299	0.4281	0.3529	0.6792	**0.0001**	0.1616

TFPI: Tissue factor pathway inhibitor; TF: Tissue factor; TF: Tissue factor; t-PA: Tissue plasminogen activator; PAI-1: Plasminogen activator inhibitor-1; significant differences are denoted by bold *p*-values.

**Table 8:** The univariate and multivariate Cox regression models for DFS regarding pre-treatment haemostatic parameters.

Variables	Univariate		Multivariate	
	HR (95% CI)	*p*-Values	HR (95% CI)	*p*-Values
Pre-treatment TFPI activity				
Low	3.20		3.21	
High	(0.41–24.97)	0.2675	(0.39–26.49)	0.2786
Pre-treatment TFPI antigen				
Low	1.96		0.51	
High	(0.57–6.72)	0.2822	(0.12–2.19)	0.3643
Pre-treatment TF antigen				
Low	4.24		4.20	
High	(0.91–19.65)	0.0649	(0.79–22.26)	0.0921
Pre-treatment TF activity				
Low	14.33		7.91	
High	(1.81–113.28)	**0.0116**	(0.87–71.74)	0.0660
Pre-treatment t-PA antigen				
Low	3.99		2.68	
High	(0.86–18.48)	0.0768	(0.53–13.61)	0.2332
Pre-treatment PAI-1 antigen				
Low	8.39		11.84	
High	(1.07–65.58)	**0.0427**	(0.49–286.23)	0.1283

TFPI: Tissue factor pathway inhibitor; TF: Tissue factor; TF: Tissue factor; t-PA: Tissue plasminogen activator; PAI-1: Plasminogen activator inhibitor-1. The Cox proportional hazards model was used for unadjusted univariate and adjusted multivariate analyses—BMI, age at the time of diagnosis, staging, intrinsic type, histological type, nodal involvement and tumour diameter; significant differences are denoted by bold *p*-values.

**Table 9:** The univariate and multivariate Cox regression models for DFS regarding post-treatment haemostatic parameters.

Variables	Univariate		Multivariate	
	HR (95% CI)	*p*-Values	HR (95% CI)	*p*-Values
Post-treatment TFPI activity				
Low	2.30		2.16	
High	(0.61–8.69)	0.2207	(0.54–8.68)	0.2768
Post-treatment TFPI antigen				
Low	3.25		3.18	
High	(0.86–12.27)	0.0822	(0.66–15.33)	0.1499
Post-treatment TF antigen				
Low	3.13		2.03	
High	(0.68–14.49)	0.1445	(0.35–11.64)	0.4262
Post-treatment TF activity				
Low	2.98		0.49	
High	(0.79–11.25)	0.1074	(0.06–3.71)	0.4895
Post-treatment t-PA antigen				
Low	11.29		2.74	
High	(1.43–89.25)	**0.0216**	(0.68–14.63)	0.9954
Post-treatment PAI-1 antigen				
Low	1.82		2.93	
High	(0.53–6.21)	0.3405	(0.68–12.59)	0.1476

TFPI: Tissue factor pathway inhibitor; TF: Tissue factor; TF: Tissue factor; t-PA: Tissue plasminogen activator; PAI-1: Plasminogen activator inhibitor-1. The Cox proportional hazards model was used for unadjusted univariate and adjusted multivariate analyses—BMI, age at the time of diagnosis, staging, intrinsic type, histological type, nodal involvement and tumour diameter; significant differences are denoted by bold *p*-values.

## Data Availability

The data presented in this study are available in this article.

## Supplementary Materials

The following supporting information can be downloaded at: https://www.mdpi.com/article/10.3390/life13051106/s1, Table S1: The hemostatic profile analysis of pre/post treatment TFPI activity, TFPI antigen and TF antigen in respect to clinicopathological features; Table S2: The hemostatic profile analysis of pre/post treatment TF activity, t-PA antigen and PAI-1 antigen in respect to clinicopathological features; Figure S1: Disease-free survival (DFS) and overall survival (OS) analysis of the studied population regarding types of adjuvant therapy in IBrC patients.
