# Supplementary material for: Hypercoagulability State Combined with Post-Treatment Hypofibrinolysis in Invasive Breast Cancer: A Seven-Year Follow-Up Evaluating Disease-Free and Overall Survival
Source: Life (Basel). 2023 Apr 28;13(5):1106. doi: 10.3390/life13051106 (PMC10222121; doi:10.3390/life13051106)
Supplement: Supplementary file 1 [file life-13-01106-s001.zip › life-2344263-supplementary.pdf]

**Table S1.** The hemostatic profile analysis of pre/post treatment TFPI activity, TFPI antigen and TF antigen in respect to clinicopathological features.

| Variables of interest | Pre-treatment TFPI activity [U/mL] *p*-value | Post-treatment TFPI activity [U/mL] *p*-value | *p*-value* | Pre-treatment TFPI antigen [ng/mL] *p*-value | Post-treatment TFPI antigen [ng/mL] *p*-value | *p*-value** | Pre-treatment TF antigen [ng/mL] *p*-value | Post-treatment TF antigen [ng/mL] *p*-value | *p*-value*** |
|---|---|---|---|---|---|---|---|---|---|
| | 1.32 | 1.72 | | 40.68 | 42.93 | | 581.87 | 837.69 | |
| Age | 1.10/1.50 | 1.30/1.96 | **0.0077** | 33.40/46.44 | 33.71/52.76 | 0.2116 | 400.86/752.35 | 689.43/999.12 | **0.0001** |
| < 56 years | 1.30 | 1.70 | | 48.24 | 42.77 | | 500.39 | 789.00 | |
| ≥ 56 years | 1.16/1.48 | 1.12/2.20 | **0.0155** | 41.00/60.32 | 38.54/52.48 | 0.3575 | 402.49/596.73 | 642.06/973.34 | **<0.0001** |
| | *p*=0.7324 | *p*=0.8877 | | ***p*=0.0052** | *p*=0.5132 | | *p*=0.2039 | *p*=0.8063 | |
| | 1.39 | 1.75 | | 42.36 | 44.30 | | 579.64 | 827.41 | |
| Menopausal status | 1.28/1.56 | 1.56/2.08 | **0.0258** | 31.96/46.44 | 33.71/46.53 | 0.1240 | 431.59/752.35 | 594.88/1002.42 | **0.0076** |
| pre-menopause | 1.28 | 1.64 | | 45.86 | 42.65 | | 521.26 | 815.96 | |
| post-menopause | 1.14/1.46 | 1.10/2.10 | **0.0045** | 39.48/60.16 | 38.13/53.46 | 0.5299 | 400.94/686.50 | 660.92/973.34 | **<0.0001** |
| | *p*=0.2901 | *p*=0.4899 | | *p*=0.0934 | *p*=0.6061 | | *p*=0.3869 | *p*=0.8544 | |
| | 1.31 | 1.70 | | 40.86 | 42.22 | | 551.95 | 805.96 | |
| Body mass index | 1.20/1.50 | 0.90/1.96 | 0.0641 | 35.04/48.24 | 33.01/47.86 | 0.6435 | 402.49/718.7 | 671.43/973.34 | **<0.0001** |
| BMI ≤ 24.99 kg/m$^2$ | 1.31 | 1.72 | | 48.02 | 44.30 | | 552.70 | 842.45 | |
| BMI ≥ 25 kg/m$^2$ | 1.1/1.48 | 1.42/2.20 | **0.0026** | 40.68/61.88 | 39.16/52.76 | 0.4653 | 400.94/726.12 | 642.06/1002.68 | **<0.0001** |
| | *p*=0.5393 | *p*=0.5058 | | ***p*=0.0467** | *p*=0.1958 | | *p*=0.9528 | *p*=0.7338 | |
| | 1.24 | 1.30 | | 40.98 | 37.93 | | 597.19 | 785.1 | 0.0747 |
| Parity status | 1.16/1.4 | 0.90/2.20 | 0.7532 | 31.96/50.4 | 33.71/42.16 | 0.7532 | 521.76/724.55 | 671.43/890.45 | |
| 0 | 1.30 | 1.74 | | 44.04 | 42.93 | | 565.46 | 837.69 | **<0.0001** |
| 1-2 | 1.14/1.46 | 1.40/2.08 | **0.0004** | 37.64/55.76 | 38.39/52.76 | 0.8105 | 402.49/726.12 | 660.85/973.34 | |
| 3 and more | 1.42 | 1.70 | | 47.68 | 44.84 | | 500.39 | 774.18 | **0.0030** |
| | 1.16/1.58 | 1.10/1.96 | 0.2489 | 40.68/61.88 | 35.44/52.48 | 0.2489 | 392.64/554.97 | 660.92/1022.32 | |
| | *p*=0.5062 | *p*=0.8238 | | *p*=0.3091 | *p*=0.5423 | | *p*=0.4857 | *p*=0.9686 | |
| | 1.42 | 1.82 | | 44.04 | 46.51 | | 596.73 | 874.53 | **0.0006** |
| Smoking status | 1.2/1.58 | 1.48/2.38 | **0.0168** | 38.00/55.76 | 42.53/52.76 | 0.5228 | 400.86/774.32 | 660.92/985.32 | |
| Smokers | 1.28 | 1.68 | | 44.48 | 41.91 | | 539.93 | 800.00 | **<0.0001** |
| Non-smokers | 1.12/1.46 | 1.10/2.00 | **0.0078** | 37.64/58.80 | 33.71/52.48 | 0.4688 | 431.59/649.85 | 660.85/999.12 | |
| | *p*=0.1864 | *p*=0.2752 | | *p*=0.8311 | *p*=0.1116 | | *p*=0.4755 | *p*=0.5170 | |
| | 1.38 | 1.92 | | 46.44 | 43.23 | | 554.97 | 756.30 | |
| Tumour localization | 1.26/1.58 | 1.4/2.38 | **0.0066** | 40.12/60.32 | 38.54/52.76 | 0.5178 | 400.94/686.50 | 615.39/999.12 | **0.0001** |
| Left breast | 1.20 | 1.56 | | 43.00 | 42.16 | | 550.43 | 866.51 | |
| Right breast | 1.12/1.40 | 1.12/1.08 | **0.0190** | 35.04/50.80 | 33.76/51.59 | 0.7213 | 402.49/726.12 | 689.43/985.32 | **<0.0001** |
| | ***p*=0.0411** | *p*=0.1711 | | *p*=0.1135 | *p*=0.4075 | | *p*=0.7843 | *p*=0.2059 | |

| | | | | | | | | | |
|---|---|---|---|---|---|---|---|---|---|
| | 1.30 | 1.74 | | 44.04 | 43.23 | | 550.43 | 822.71 | |
| Histological type | 1.12/1.481.32 | 1.12/2.20 | **0.0003** | 38.00/58.80 | 37.87/56.46 | 0.9701 | 392.64/654.26 | 660.92/985.32 | **<0.0001** |
| IDC | 1.26/1.60 | 1.68 | | 44.48 | 42.53 | | 686.50 | 884.08 | |
| ILC | *p*=0.1991 | 1.18/1.76 | 0.5940 | 35.60/50.40 | 35.44/45.66 | 0.4413 | 520.76/728.94 | 642.06/1002.68 | 0.0506 |
| | | *p*=0.3461 | | *p*=0.7561 | *p*=0.4315 | | *p*=0.2636 | *p*=0.7719 | |
| | 1.32 | 1.60 | | 44.48 | 42.61 | | 554.97 | 831.91 | |
| Histological grade | 1.16/1.56 | 1.10/2.00 | **0.0194** | 37.12/55.76 | 33.76/52.48 | 0.7190 | 402.49/726.12 | 660.85/1002.42 | **<0.0001** |
| G1 + G2 | 1.28 | 1.96 | | 43.44 | 46.50 | | 474.14 | 800.00 | |
| G3 | 0.84/1.42 | 1.76/2.52 | **0.0046** | 40.28/58.80 | 39.16/52.76 | 0.7007 | 386.02/592.28 | 712.42/847.20 | 0.0107 |
| | *p*=0.1811 | ***p*=0.0342** | | *p*=0.8929 | *p*=0.4091 | | *p*=0.4897 | *p*=0.6154 | |
| | 1.34 | 1.72 | | 44.48 | 42.28 | | 539.93 | 774.18 | |
| cT category (7th ed.) | 1.12/1.60 | 1.02/2.20 | **0.0171** | 38.00/50.80 | 36.06/56.46 | 0.8741 | 402.49/724.55 | 615.39/1002.68 | **<0.0001** |
| T1 | 1.30 | 1.70 | | 44.16 | 43.76 | | 562.47 | 837.69 | |
| T2 | 1.16/1.42 | 1.18/1.96 | **0.0032** | 37.64/60.36 | 38.54/49.00 | 0.8552 | 400.94/654.26 | 738.68/900.71 | **0.0001** |
| | *p*=0.4889 | *p*=0.8255 | | *p*=0.7611 | *p*=0.9273 | | *p*=0.9818 | *p*=0.7727 | |
| | 1.34 | 1.58 | | 43.74 | 42.92 | | 505.96 | 805.96 | |
| cN category (7th ed.) | 1.16/1.56 | 0.90/1.96 | **0.0482** | 39.08/60.16 | 37.87/51.59 | 0.6392 | 392.64/686.50 | 655.33/985.32 | **<0.0001** |
| N0 | 1.21 | 1.81 | | 45.80 | 42.85 | | 594.51 | 865.64 | |
| N1 | 1.12/1.46 | 1.56/2.52 | **0.0007** | 32.24/55.00 | 36.06/58.16 | 0.6475 | 500.39/728.94 | 691.40/1121.33 | **0.0012** |
| | *p*=0.2796 | ***p*=0.0405** | | *p*=0.7839 | *p*=0.9550 | | *p*=0.1050 | *p*=0.3881 | |
| | 1.39 | 1.48 | | 42.68 | 42.39 | | 459.23 | 743.30 | |
| cStage (7th ed.) | 1.05/1.61 | 0.70/2.15 | 0.3037 | 38.54/50.02 | 36.66/54.47 | 0.9544 | 386.02/721.63 | 603.96/1002.55 | **0.0004** |
| IA | 1.29 | 1.76 | | 45.56 | 43.08 | | 564.71 | 834.90 | |
| IIA+IIB | 1.15/1.44 | 1.47/2.07 | **0.0001** | 36.84/60.26 | 37.23/52.18 | 0.8137 | 435.58/690.19 | 690.42/925.19 | **<0.0001** |
| | *p*=0.3340 | *p*=0.1047 | | *p*=0.5312 | *p*=0.9459 | | *p*=0.2515 | *p*=0.6782 | |
| | 1.29 | 1.64 | | 44.58 | 42.65 | | 556.45 | 815.96 | |
| Molecular subtype | 1.14/1.58 | 1.30/2.00 | **0.0225** | 38.00/50.80 | 35.44/48.85 | 0.5214 | 402.49/728.94 | 642.06/985.32 | **<0.0001** |
| Luminal A | 1.34 | 1.87 | | 42.90 | 44.80 | | 538.37 | 827.41 | |
| Non-luminal A | 1.14/1.42 | 1.10/.38 | **0.0046** | 37.64/61.88 | 38.93/56.46 | 0.7702 | 392.64/654.26 | 691.40/999.12 | **0.0001** |
| | *p*=0.7540 | *p*=0.2761 | | *p*=0.6871 | *p*=0.3435 | | *p*=0.6926 | *p*=0.9940 | |
| | 1.28 | 1.50 | | 44.68 | 42.16 | | 550.43 | 831.91 | |
| Proliferation marker | 1.16/1.5 | 0.76/1.96 | 0.1587 | 38.00/55.00 | 33.71/47.86 | 0.2953 | 400.94/726.12 | 660.85/1002.68 | **<0.0001** |
| expression | 1.36 | 1.94 | | 42.36 | 46.99 | | 554.97 | 822.71 | |
| Ki67 < 20% | 1.10/1.48 | 1.68/2.52 | **0.0003** | 37.64/61.88 | 41.41/56.46 | 0.3392 | 439.56/581.87 | 691.40/890.45 | **0.0005** |
| Ki67 ≥ 20% | *p*=0.9938 | ***p*=0.0042** | | *p*=0.9198 | *p*=0.0546 | | *p*=0.7982 | *p*=0.6756 | |

TFPI: Tissue factor pathway inhibitor; TF: Tissue factor; IDC: Invasive ductal carcinoma; ILC: Invasive lobular carcinoma; G1: low grade; G2: intermediate grade; G3: high grade; T1: tumor diameter ≤ 2 cm; T2: tumor diameter > 2 cm to ≤ 5 cm; N0: lack of lymph node metastases; N1: spread to axillary lymph nodes; ER: oestrogen receptor; PR: progesterone receptor; HER2: human epidermal growth factor receptor 2; Ki67: proliferation marker; BCS: breast-conserving surgery; MRM: modified radical mastectomy. Data are expressed as median (Me) and the inter-quartile range (IQR) [lower quartile (Q1)/upper quartile (Q3)]; *p*-value* for differences between pre-treatment and post-treatment TFPI activity; *p*-value** for differences between pre-treatment and post-treatment TFPI antigen; *p*-value*** for differences between pre-treatment and post-treatment TF antigen; significant differences are denoted by bold *p*-values.

**Table S2.** The hemostatic profile analysis of pre/post treatment TF activity, t-PA antigen and PAI-1 antigen in respect to clinicopathological features.

| **Variables of interest** | **Pre-treatment TF activity** [U/mL] *p*-value | **Post-treatment TF activity** [U/mL] *p*-value | ***p*-value*** | **Pre-treatment t-PA antigen** [ng/mL] *p*-value | **Post-treatment t-PA antigen** [ng/mL] *p*-value | ***p*-value**** | **Pre-treatment PAI-1 antigen** [ng/mL] *p*-value | **Post-treatment PAI-1 antigen** [ng/mL] *p*-value | ***p*-value***** |
|---|---|---|---|---|---|---|---|---|---|
| | 13.88 | 18.64 | **0.1128** | 5.30 | 3.13 | **0.0003** | 38.56 | 77.05 | **0.0004** |
| Age | 11.62/29.88 | 11.20/38.53 | | 4.23/6.87 | 1.79/4.21 | | 27.73/49.60 | 44.20/84.33 | |
| < 56 years | 11.86 | 27.69 | **0.0010** | 6.16 | 3.01 | **0.0021** | 37.19 | 79.20 | **<0.0001** |
| ≥ 56 years | 10.42/25.88 | 12.76/34.09 | | 4.30/7.16 | 2.05/5.91 | | 30.30/44.46 | 49.87/85.97 | |
| | *p*=0.1935 | *p*=0.7437 | | *p*=0.5037 | *p*=0.3806 | | *p*=0.5424 | *p*=0.3231 | |
| Menopausal | 13.46 | 16.78 | 0.0962 | 5.20 | 2.55 | **0.0186** | 36.83 | 81.20 | **0.0015** |
| status | 10.61/23.59 | 11.22/32.97 | | 4.23/7.72 | 2.02/4.21 | | 26.42/49.60 | 47.33/85.08 | |
| pre-menopause | 12.605 | 27.84 | **0.0020** | 5.84 | 3.21 | **0.0001** | 38.23 | 77.48 | **<0.0001** |
| post-menopause | 11.17/29.59 | 12.76/37.68 | | 4.28/7.12 | 1.98/4.96 | | 30.30/44.97 | 49.1/85.97 | |
| | *p*=0.7864 | *p*=0.4736 | | *p*=0.9234 | *p*=0.4682 | | *p*=0.8339 | *p*=0.7731 | |
| | 13.65 | 26.15 | **0.0041** | 5.26 | 2.85 | **0.0014** | 37.03 | 55.30 | **0.0024** |
| Body mass index | 11.62/29.59 | 12.12/37.68 | | 4.14/6.7 | 2.18/4.96 | | 27.73/45.48 | 45.96/79.31 | |
| BMI ≤ 24.99 kg/m$^2$ | 12.085 | 27.41 | **0.0218** | 6.10 | 3.21 | **0.0005** | 38.54 | 82.05 | **<0.0001** |
| BMI ≥ 25 kg/m$^2$ | 10.03/23.31 | 11.18/34.09 | | 4.34/7.25 | 1.87/4.94 | | 30.3/45.3 | 73.27/87.71 | |
| | *p*=0.1714 | *p*=0.5542 | | *p*=0.2804 | *p*=0.8418 | | *p*=0.9176 | ***p*=0.0021** | |
| | 17.705 | 28.16 | | 6.065 | 3.4 | 0.1730 | 33.48 | 54.03 | |
| Parity status | 12.31/39.16 | 13.17/43.56 | 0.1159 | 3.86/8.82 | 2.18/6.42 | | 22.66/40.00 | 45.96/78.15 | **0.0277** |
| 0 | 12.75 | 17.91 | | 5.3 | 2.69 | **<0.0001** | 38.52 | 79.31 | |
| 1-2 | 10.61/24.17 | 11.22/33.11 | **0.0028** | 4.30/6.86 | 1.98/4.21 | | 30.27/47.43 | 49.1/84.65 | **<0.0001** |
| 3 and more | 12.46 | 27.99 | | 6.13 | 4.21 | 0.3109 | 37.9 | 78.46 | |
| | 11.47/33.47 | 20.3/38.1 | 0.1330 | 4.2/7.16 | 2.05/6.99 | | 33.7/45.3 | 49.48/87.58 | **0.0015** |
| | *p*=0.4813 | *p*=0.4918 | | *p*=0.9310 | *p*=0.4127 | | *p*=0.5833 | *p*=0.4811 | |
| | 11.77 | 27.13 | **0.0395** | 6.66 | 3.28 | **0.0217** | 35.50 | 79.20 | **0.0049** |
| Smoking status | 9.81/23.59 | 16.10/34.09 | | 4.30/7.16 | 2.68/4.21 | | 33.70/42.73 | 49.10/87.58 | |
| Smokers | 13.60 | 27.13 | **0.0079** | 5.48 | 2.67 | **0.0001** | 38.52 | 78.15 | **<0.0001** |
| Non-smokers | 11.17/29.59 | 11.18/37.68 | | 4.23/7.12 | 1.78/5.02 | | 28.10/47.43 | 47.70/84.65 | |
| | *p*=0.4704 | *p*=0.4908 | | *p*=0.3091 | *p*=0.1893 | | *p*=0.7182 | *p*=0.7803 | |
| Tumour | 11.77 | 27.99 | **0.0011** | 5.96 | 3.13 | **0.0007** | 42.73 | 79.44 | **0.0001** |
| localization | 10.03/29.59 | 12.12/38.53 | | 4.28/7.16 | 2.02/4.96 | | 33.96/49.82 | 49.1/85.08 | |
| Left breast | 13.70 | 18.64 | 0.0856 | 5.30 | 2.69 | **0.0010** | 35.12 | 73.27 | **<0.0001** |
| Right breast | 12.31/25.88 | 11.20/33.45 | | 4.14/7.12 | 1.98/4.55 | | 24.18/40.44 | 47.33/85.29 | |

| | | | | | | | | | |
|---|---|---|---|---|---|---|---|---|---|
| | *p*=0.2485 | *p*=0.4201 | | *p*=0.9410 | *p*=0.8244 | | ***p*=0.0210** | *p*=0.4074 | |
| | 13.16 | 27.13 | **0.0011** | 5.87 | 3.34 | **0.0001** | 39.39 | 79.31 | **<0.0001** |
| Histological type | 10.61/29.59 | 12.06/38.53 | | 4.30/7.16 | 2.02/5.09 | | 32.37/47.36 | 49.87/86.65 | |
| IDC | 12.31 | 27.13 | 0.1097 | 4.83 | 2.31 | **0.0077** | 28.10 | 47.70 | **0.0077** |
| ILC | 11.80/21.31 | 11.22/32.97 | | 3.86/6.16 | 1.78/2.57 | | 22.66/32.37 | 45.96/79.20 | |
| | *p*=0.8684 | *p*=0.5551 | | *p*=0.2380 | ***p*=0.0193** | | ***p*=0.0199** | *p*=0.0857 | |
| | 12.46 | 25.17 | **0.0011** | 5.69 | 3.14 | **0.0001** | 37.19 | 77.90 | **<0.0001** |
| Histological grade | 10.61/26.92 | 12.06/37.68 | | 4.207.12 | 2.18/5.09 | | 27.73/44.97 | 46.21/85.97 | |
| G1 + G2 | 14.18 | 27.69 | 0.1159 | 5.71 | 2.31 | **0.0046** | 40.44 | 81.25 | **0.0019** |
| G3 | 11.86/24.17 | 10.13/31.99 | | 5.10/7.25 | 1.98/3.95 | | 30.30/49.06 | 72.58/84.33 | |
| | *p*=0.4091 | *p*=0.7672 | | *p*=0.8225 | *p*=0.2363 | | *p*=0.3237 | *p*=0.3746 | |
| cT category (7th | 12.31 | 27.13 | **0.0030** | 5.26 | 2.67 | **0.0001** | 38.56 | 74.69 | **0.0001** |
| ed.) | 11.47/24.17 | 12.76/34.09 | | 4.23/6.34 | 1.66/4.21 | | 27.73/47.36 | 47.70/84.33 | |
| T1 | 13.70 | 27.13 | **0.0416** | 6.44 | 3.58 | **0.0074** | 37.20 | 79.20 | **0.0001** |
| T2 | 9.02/29.59 | 11.22/42.63 | | 4.55/7.78 | 2.49/6.99 | | 32.37/44.60 | 60.72/87.12 | |
| | *p*=0.8433 | *p*=0.8672 | | *p*=0.1246 | *p*=0.0704 | | *p*=0.9939 | *p*=0.3420 | |
| cN category (7th | 12.61 | 27.84 | **0.0009** | 5.70 | 3.31 | **0.0012** | 37.73 | 78.18 | **<0.0001** |
| ed.) | 10.61/24.17 | 12.12/38.53 | | 4.23/7.38 | 2.05/5.02 | | 28.10/47.36 | 49.48/85.29 | |
| N0 | 13.52 | 17.51 | 0.1701 | 5.68 | 2.64 | **0.0003** | 37.88 | 76.68 | **0.0002** |
| N1 | 11.77/29.88 | 11.20/32.97 | | 4.83/6.34 | 1.98/4.21 | | 30.30/45.30 | 46.21/84.65 | |
| | *p*=0.5398 | *p*=0.2870 | | *p*=0.8909 | *p*=0.2981 | | *p*=0.8339 | *p*=0.8026 | |
| | 12.06 | 29.09 | **0.0018** | 4.92 | 2.98 | **0.0119** | 36.70 | 76.30 | **0.0066** |
| cStage (7th ed.) | 11.10/23.88 | 15.41/38.11 | | 4.22/6.76 | 1.69/4.75 | | 27.92/47.74 | 48.59/84.71 | |
| IA | 13.65 | 17.51 | 0.0638 | 6.10 | 3.07 | **0.0001** | 38.04 | 78.83 | **<0.0001** |
| IIA+IIB | 11.12/29.74 | 11.25/33.77 | | 4.45/7.43 | 2.18/5.47 | | 31.34/44.95 | 47.02/85.97 | |
| | *p*=0.3733 | *p*=0.3897 | | *p*=0.1918 | *p*=0.5115 | | *p*=0.9579 | *p*=0.7172 | |
| Molecular | 12.06 | 17.87 | **0.0038** | 5.19 | 2.64 | **0.0001** | 34.69 | 79.26 | **<0.0001** |
| subtype | 10.03/23.31 | 11.18/34.09 | | 4.20/7.16 | 1.98/4.55 | | 27.30/43.77 | 46.21/85.97 | |
| Luminal A | 14.59 | 27.84 | **0.0490** | 5.97 | 3.64 | **0.0153** | 40.62 | 77.60 | **0.0006** |
| Non-luminal A | 11.86/39.16 | 15.65/41.89 | | 4.34/6.87 | 2.05/5.09 | | 36.46/49.60 | 49.48/84.33 | |
| | ***p*=0.0119** | *p*=0.1943 | | *p*=0.7940 | *p*=0.3667 | | *p*=0.0654 | *p*=0.9821 | |
| Proliferation | 12.31 | 20.30 | **0.0003** | 5.26 | 2.67 | **<0.0001** | 35.50 | 77.90 | **<0.0001** |
| marker expression | 10.39/23.31 | 11.22/34.09 | | 4.14/7.16 | 1.87/4.55 | | 27.30/44.60 | 46.21/85.29 | |
| Ki67 < 20% | 15.20 | 27.99 | 0.2736 | 6.06 | 3.69 | **0.0326** | 40.80 | 78.46 | **0.0019** |
| Ki67 ≥ 20% | 11.86/39.16 | 16.10/39.87 | | 4.34/6.87 | 2.60/5.09 | | 36.46/49.60 | 71.24/84.33 | |
| | ***p*=0.0126** | *p*=0.5404 | | *p*=0.5611 | *p*=0.1825 | | *p*=0.0882 | *p*=0.4293 | |

TF: Tissue factor; t-PA: Tissue plasminogen activator; PAI-1: Plasminogen activator inhibitor-1; IDC: Invasive ductal carcinoma; ILC: Invasive lobular carcinoma; G1: low grade; G2: intermediate grade; G3: high grade; T1: tumor diameter ≤ 2 cm; T2: tumor diameter > 2 cm to ≤ 5 cm; N0: lack of lymph node metastases; N1: spread to axillary lymph nodes; ER: oestrogen receptor; PR: progesterone receptor; HER2: human epidermal growth factor receptor 2; Ki67: proliferation marker; BCS: breast-conserving surgery; MRM: modified radical mastectomy. Data are expressed as median (Me) and the inter-quartile range (IQR) [lower quartile (Q1)/upper quartile (Q3)]; )]; *p*-value* for differences between pre-treatment and post-treatment TF activity; *p*-value** for differences between pre-treatment and post-treatment t-PA antigen; *p*-value*** for differences between pre-treatment and post-treatment PAI-1 antigen; significant differences are denoted by bold *p*-values.

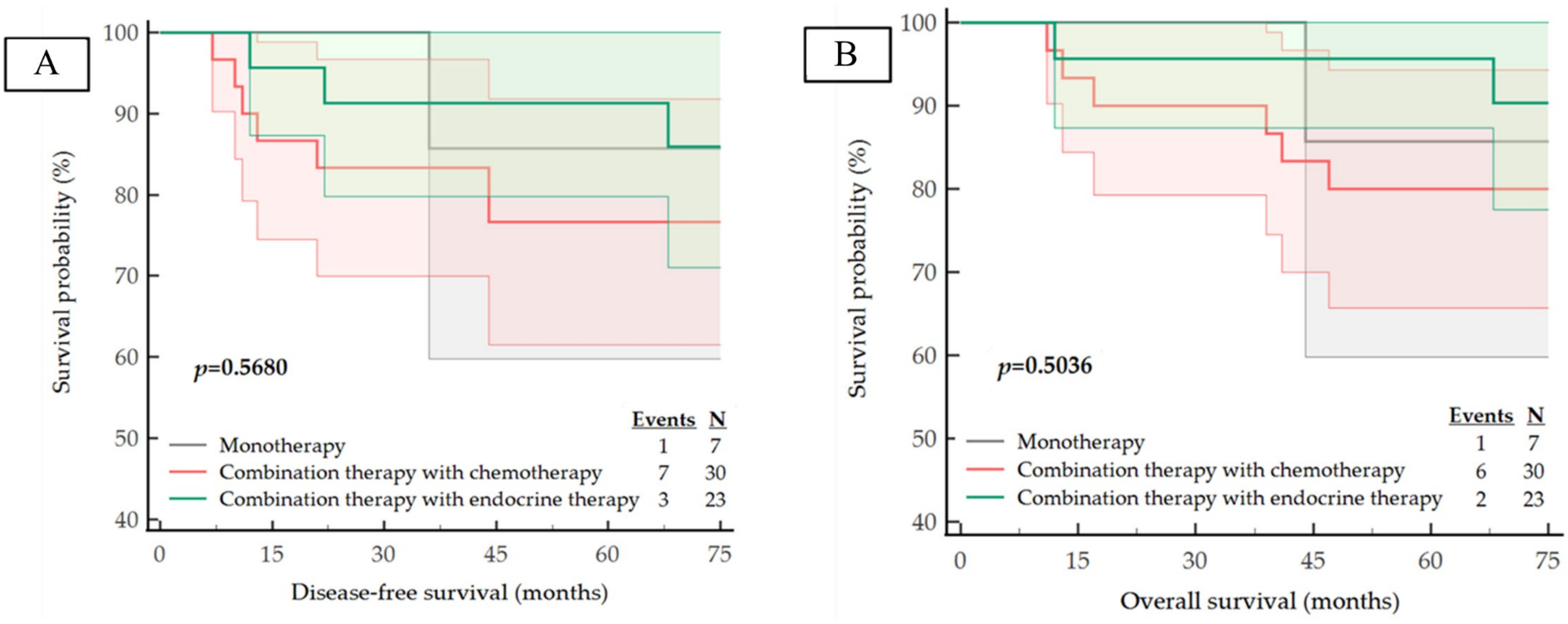


**Figure S1**. **(A)** Disease-free survival (DFS) and **(B)** overall survival (OS) analysis of the studied population regarding types of adjuvant therapy in IBrC patients.
